# Supplementary material for: Improved pearl millet genomes representing the global heterotic pool offer a framework for molecular breeding applications
Source: Commun Biol. 2023 Sep 4;6:902. doi: 10.1038/s42003-023-05258-3 (PMC10477261; doi:10.1038/s42003-023-05258-3)
Supplement: Supplementary file 2 — Supplementary information [file 42003_2023_5258_MOESM2_ESM.pdf]

Supplementary Information

Improved pearl millet genomes representing the global heterotic pool offer a framework for molecular breeding applications

Punna Ramu[1], Rakesh K. Srivastava[2*], Abhijit Sanyal[1], Kevin Fengler[3], Jun Cao[3], Yun Zhang[3], Mitali Nimkar[1], Justin Gerke[3], Sriram Shreedharan[3], Victor Llaca[3], Gregory May[3], Brooke Peterson-Burch[3], Haining Lin[3,4], Matthew King[3,5], Sayan Das[1], Vaid Bhupesh[1], Ajin Mandaokar[1], Karunakaran Maruthachalam[1], Pobbathi Krishnamurthy[1], Harish Gandhi[2,6], Abhishek Rathore[2,7], Rajeev Gupta[2,8], Annapurna Chitikineni[2,9], Prasad Bajaj[2], SK Gupta[2], C. Tara Satyavathi[10], Anand Pandravada[1], Rajeev K. Varshney[2,9*], Raman Babu[1*]

[1]Corteva Agriscience, Hyderabad, Telangana, India.
[2]International Crops Research Institute for the Semi-Arid Tropics, Hyderabad, Telangana, India.
[3]Corteva Agriscience, Johnston, IA 50131, USA.
[4]Present address: Moderna, 200 Technology Square, Cambridge, Massachusetts 02139, USA.
[5]Present address: Natera Inc., San Carlos, CA 94070, USA.
[6]Present address: International Maize and Wheat Improvement Center (CIMMYT), Nairobi, Kenya.
[7]Present address: International Maize and Wheat Improvement Center (CIMMYT), Hyderabad, India.
[8]Present address: Cereal Crops Research Unit, Edward T. Schafer Agricultural Research Center, USDA-ARS, Fargo, ND 58102, USA.
[9]Present Address: Centre for Crop & Food Innovation, State Agricultural Biotechnology Centre, Food Futures Institute, Murdoch University, Murdoch, WA 6150, Australia.
[10]Indian Council of Agricultural Research – All India Coordinated Research Project on Pearl Millet, Jodhpur, India.
[*]Corresponding authors: rajeev.varshney@murdoch.edu.au, r.k.srivastava@CGIAR.ORG, raman.babu@corteva.com

Supplementary Figure 1:

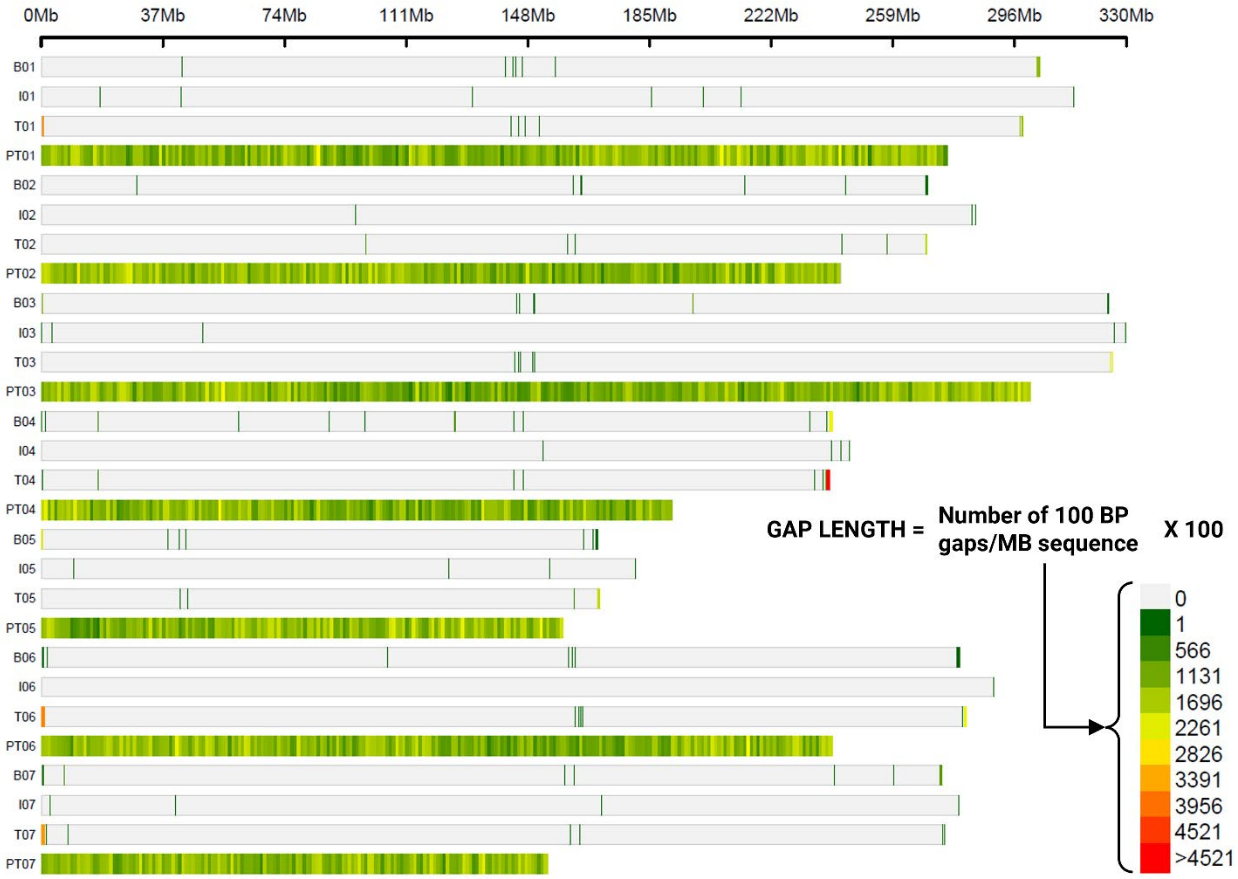

Supplementary Figure 1. Comparative gap density of four millet genomes. The number of 100 bp gaps per MB of chromosomal length is plotted for 843B (B), ICMR06777 (I), Corteva Tift Assembly (T) and Tift-2017 Assembly (PT). While each colored line is a 100 bp gap, the color signifies the number of such gaps occurring either consecutively or in proximity to each other. Consecutive gaps lead to increase in width of the line and a color change. Thus, dark green is a gap of length between 100 to 56500 bp. Yellow is a gap of length between 282600 - 339000 bp, while red is a gap of length >452100 bp. Tift-2017 has more 100 bp gaps than the other assemblies and therefore has shorter length of chromosomes.

Supplementary Figure 2:

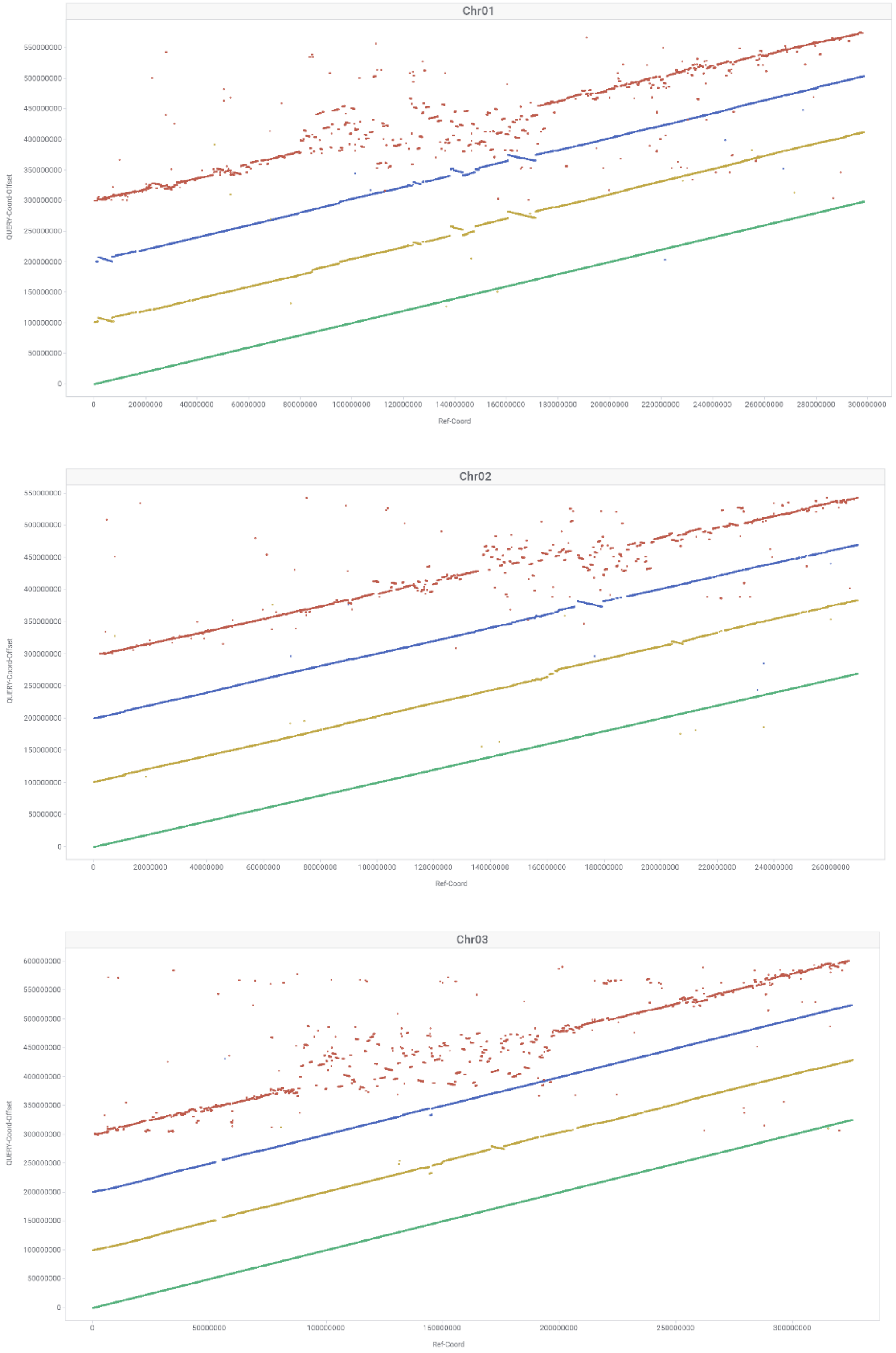

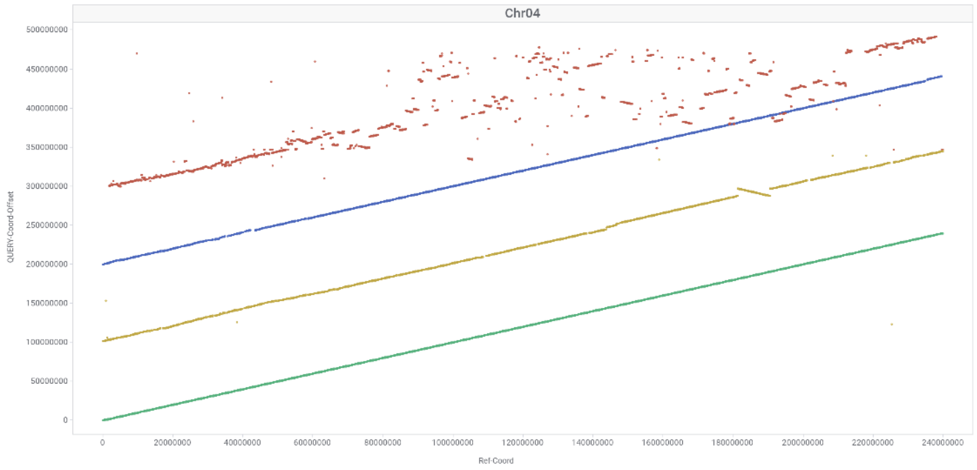
Chr04
QUERY-Coord-Offset
Ref-Coord

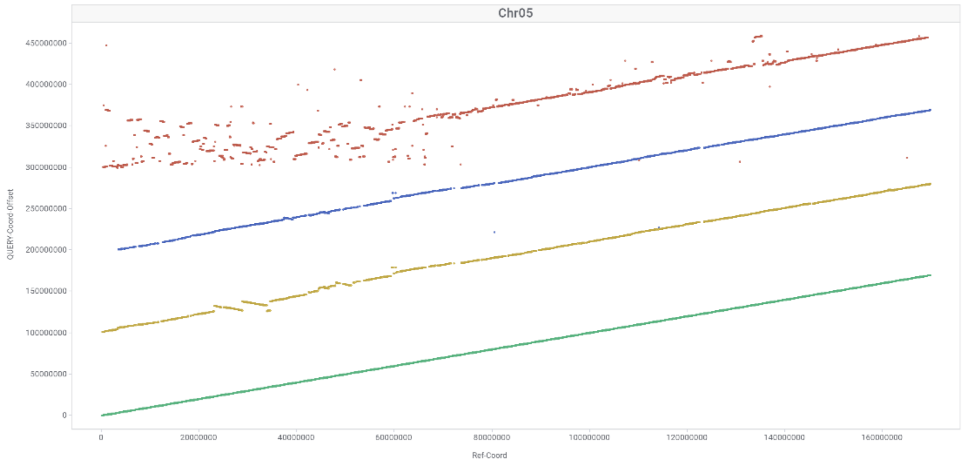
Chr05
QUERY-Coord-Offset
Ref-Coord

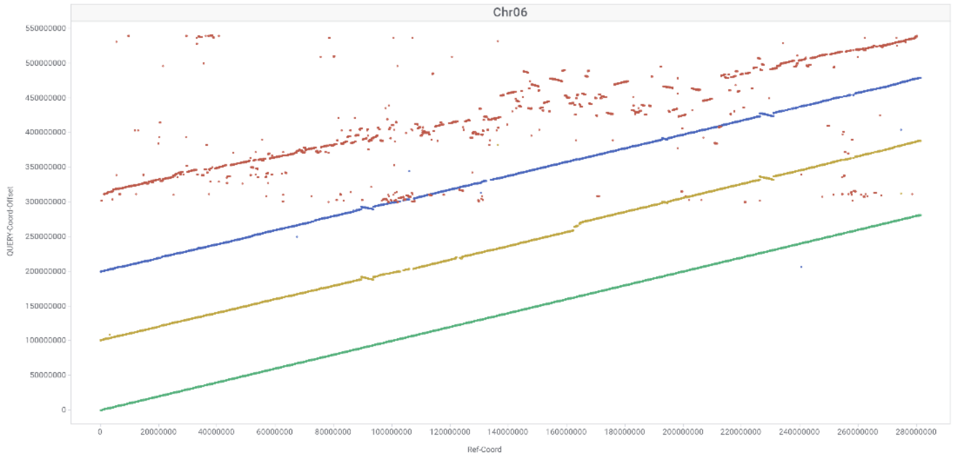
Chr06
QUERY-Coord-Offset
Ref-Coord

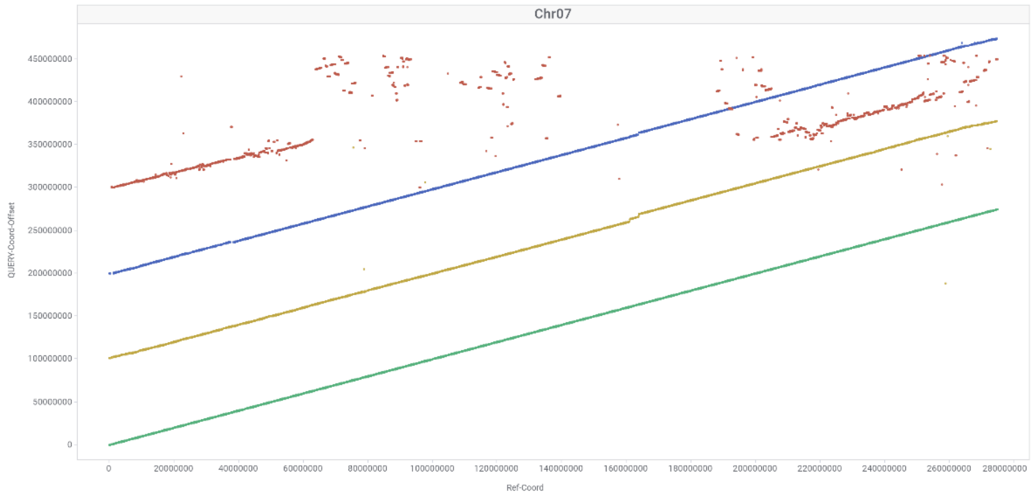

Supplementary Figure 2: Comparing the genome assemblies of Tift (green), ICMR 06777 (yellow), 843 B (blue) and Tift-2017 (red). These TagDots highlighting the structural variation among three genomes and showing the contiguity and alignment in pericentric regions.

Supplementary Figure 3:

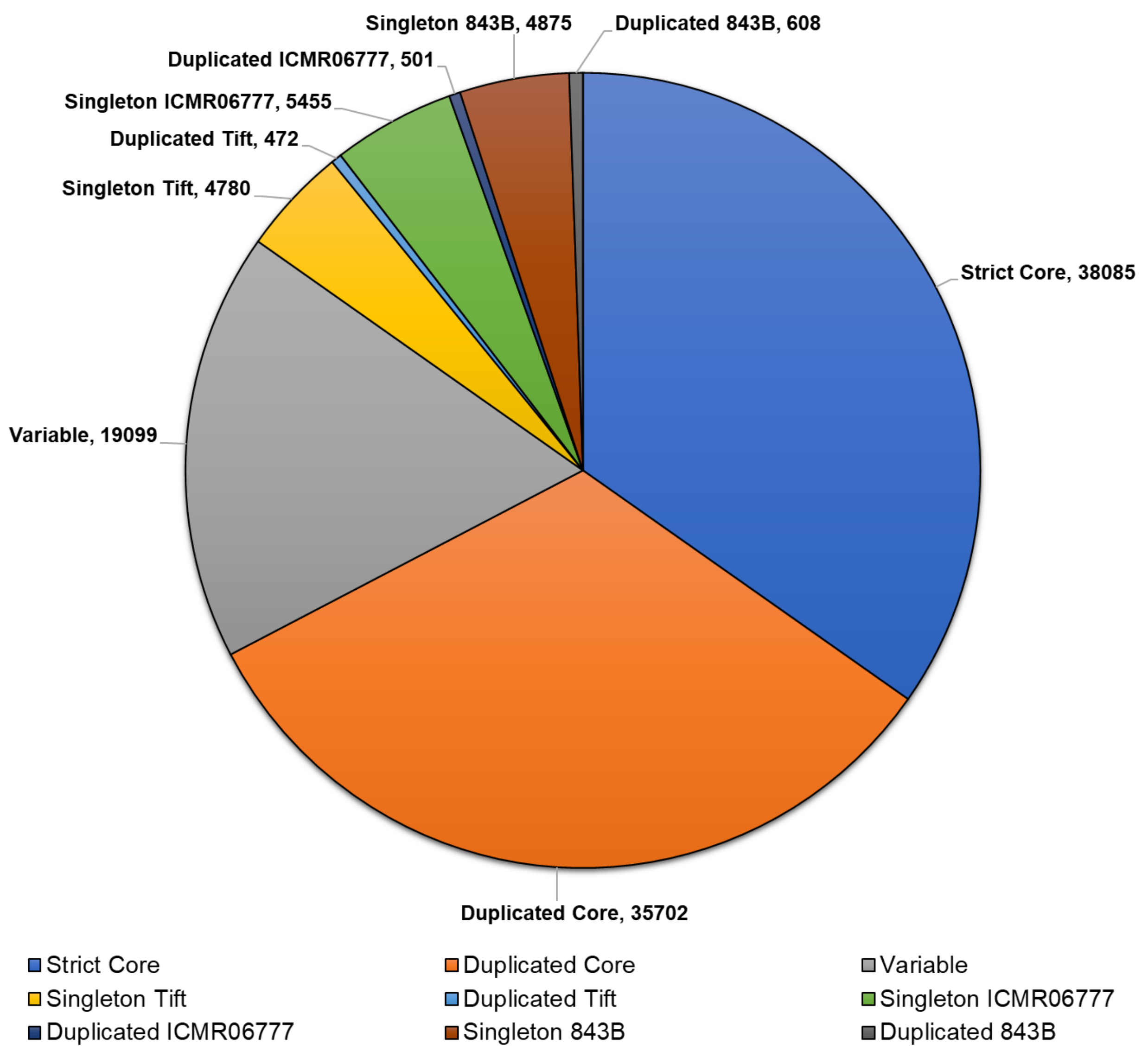

Supplementary Figure 3: Gene distribution in core, variable, and singleton clusters for assembled millet genomes.

Supplementary Figure 4:

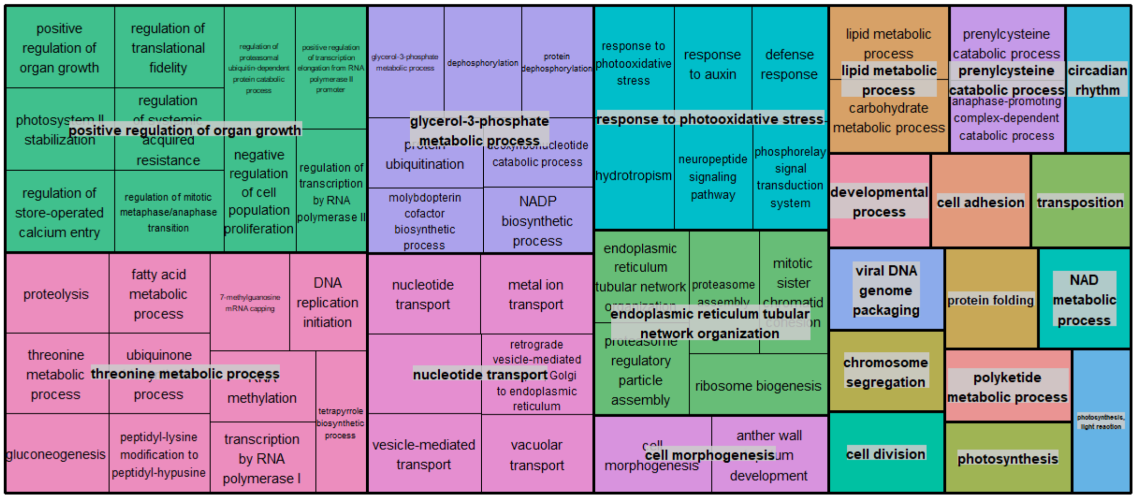

Supplementary Fig 4a. Distribution of the GO annotations for biological processes in 790 unique genes of the new Tift assembly

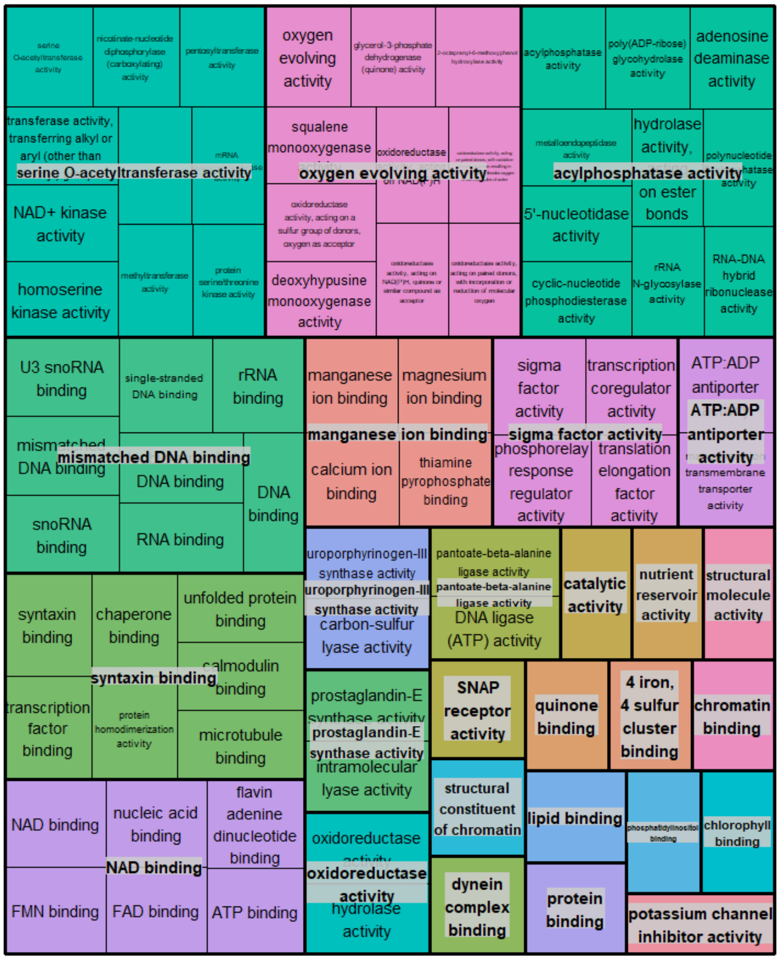

Supplementary Fig 4b. Distribution of the GO annotations for molecular function in 790 unique genes of the new Tift assembly

Supplementary Figure 5:

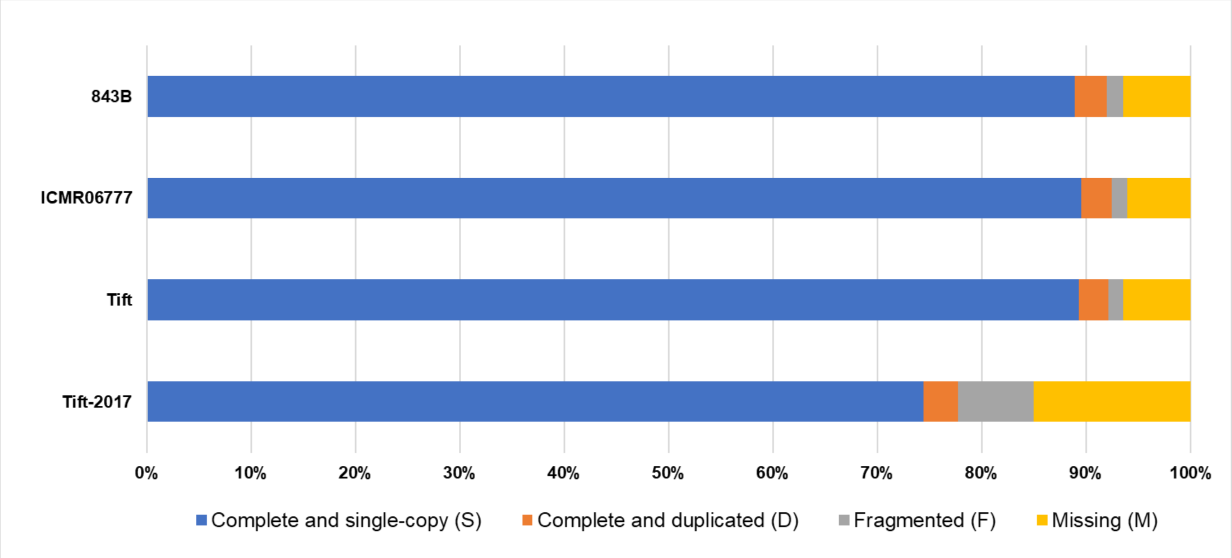

Supplementary Figure 5. Comparison of gene annotation BUSCO Scores. 4896 Poales BUSCO's are assessed for their coverage in the protein sets after gene annotation from four millet assemblies. The Tift, ICMR06777 and 843B protein sets showed an ~92% complete coverage of BUSCO's compared to ~78% by Tift-2017.

Supplementary Figure 6:

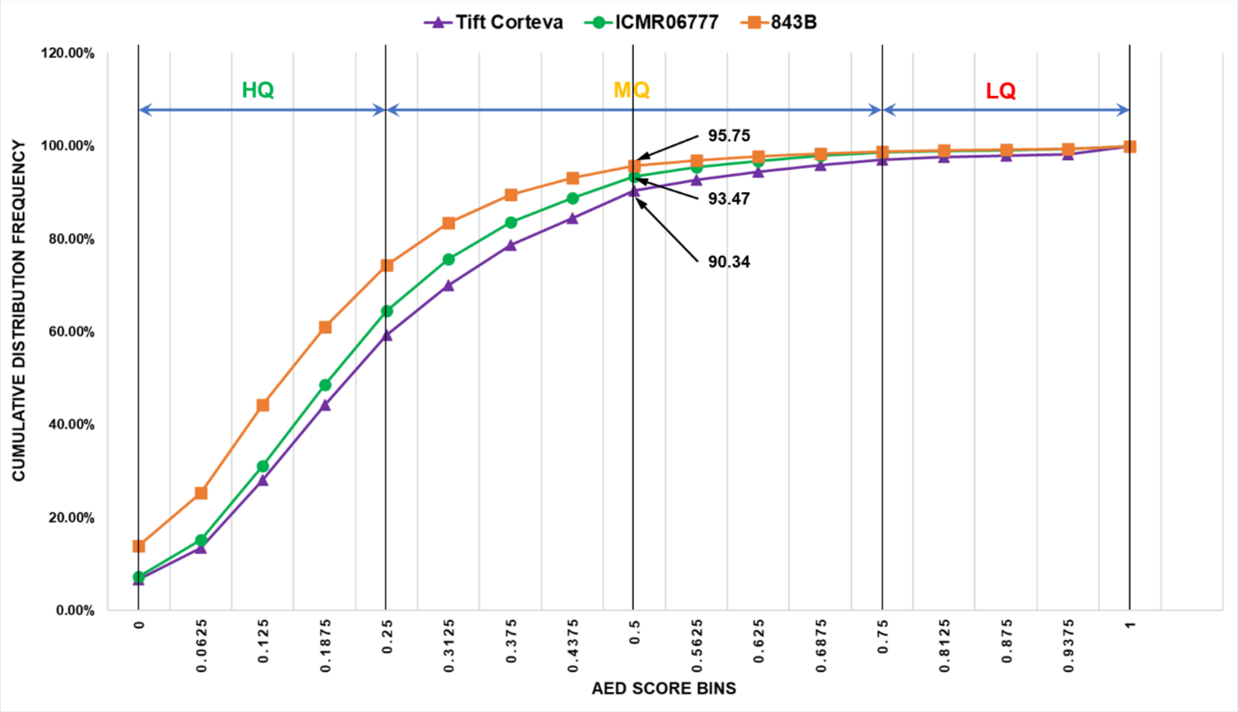

Supplementary Figure 6. Distribution of transcript AED scores for Tift, ICMR06777 and 843B. **HQ** = High-Quality, **MQ** = Medium-Quality, **LQ** = Low-Quality. Typically, HQ transcripts are very well evidence supported and rarely require manual curation to correct their gene models. MQ transcripts require manual curation however, curation complexity decreases as the AED value approaches 0.25 and >90% of the transcripts have a corrected gene model after structure curation. LQ transcripts receive the least amount of evidence support and require an extensive curation effort to correct their gene models.

Supplementary Figure 7:

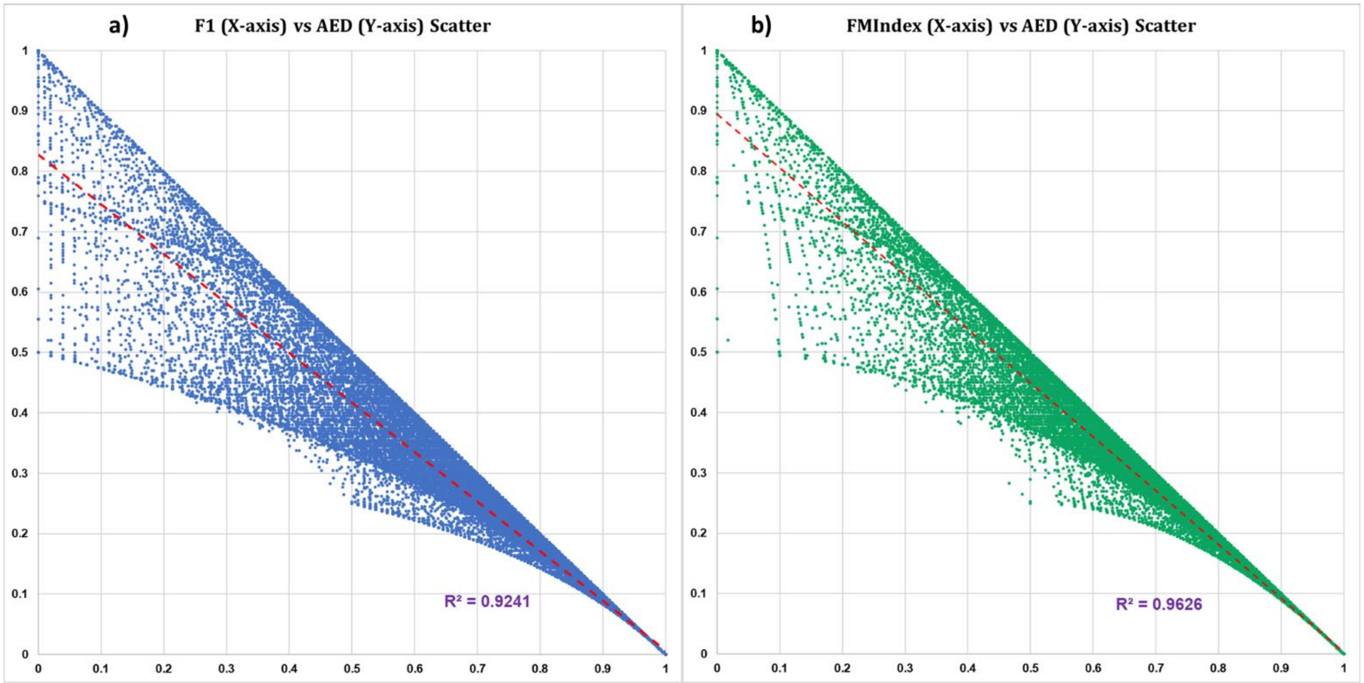

Supplementary Figure 7. a) Correlation of AED and F1 metrics. b) Correlation of AED and F-M index. While AED measures the level of dissimilarity between the final gene model structure and the alignment of evidence (cDNA or protein), the F1 and F-M indices measure the level of congruency between the two. These measures are sensitive to the number of true exon predictions and therefore are better estimates of accuracy in predictions. AED is very strongly inversely correlated to the F1 measure and to the F-M index (AED/F1 = -0.9241 and AED/F-M = -0.9626) suggesting that the support is well segregated for each transcript and there is less overlap between evidence from adjoining transcripts.

Supplementary Figure 8:

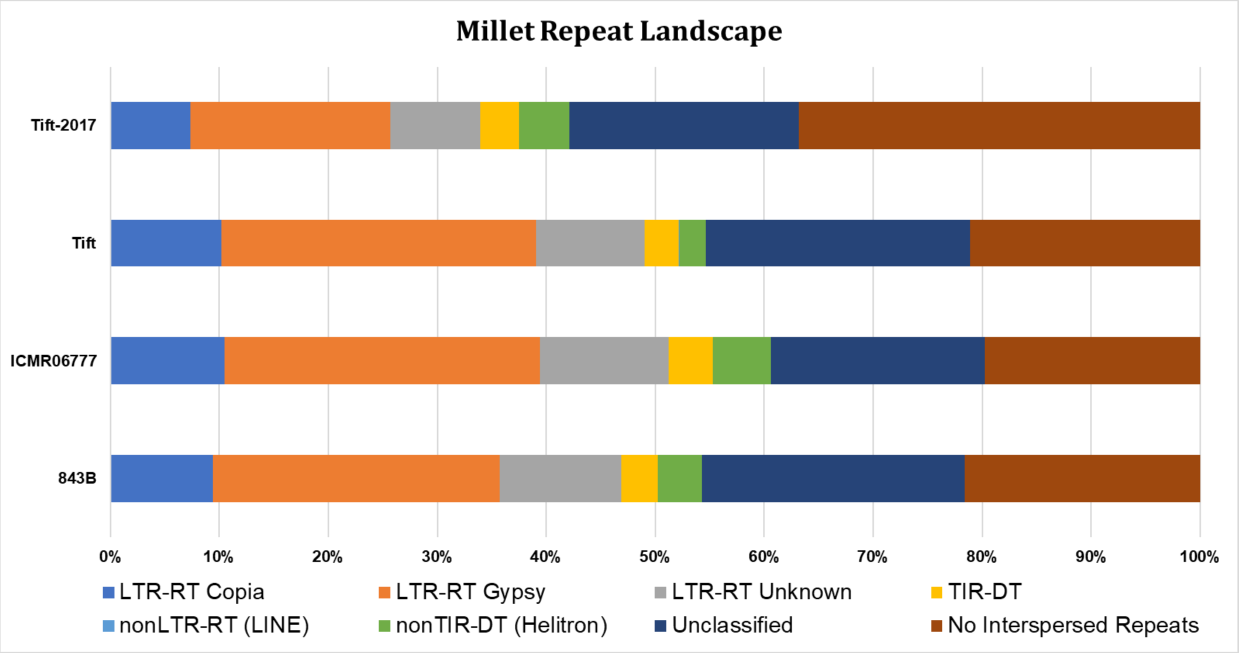

Supplementary Figure 8: Comparative repeat landscape of the millet lines.

Supplementary Figure 9:

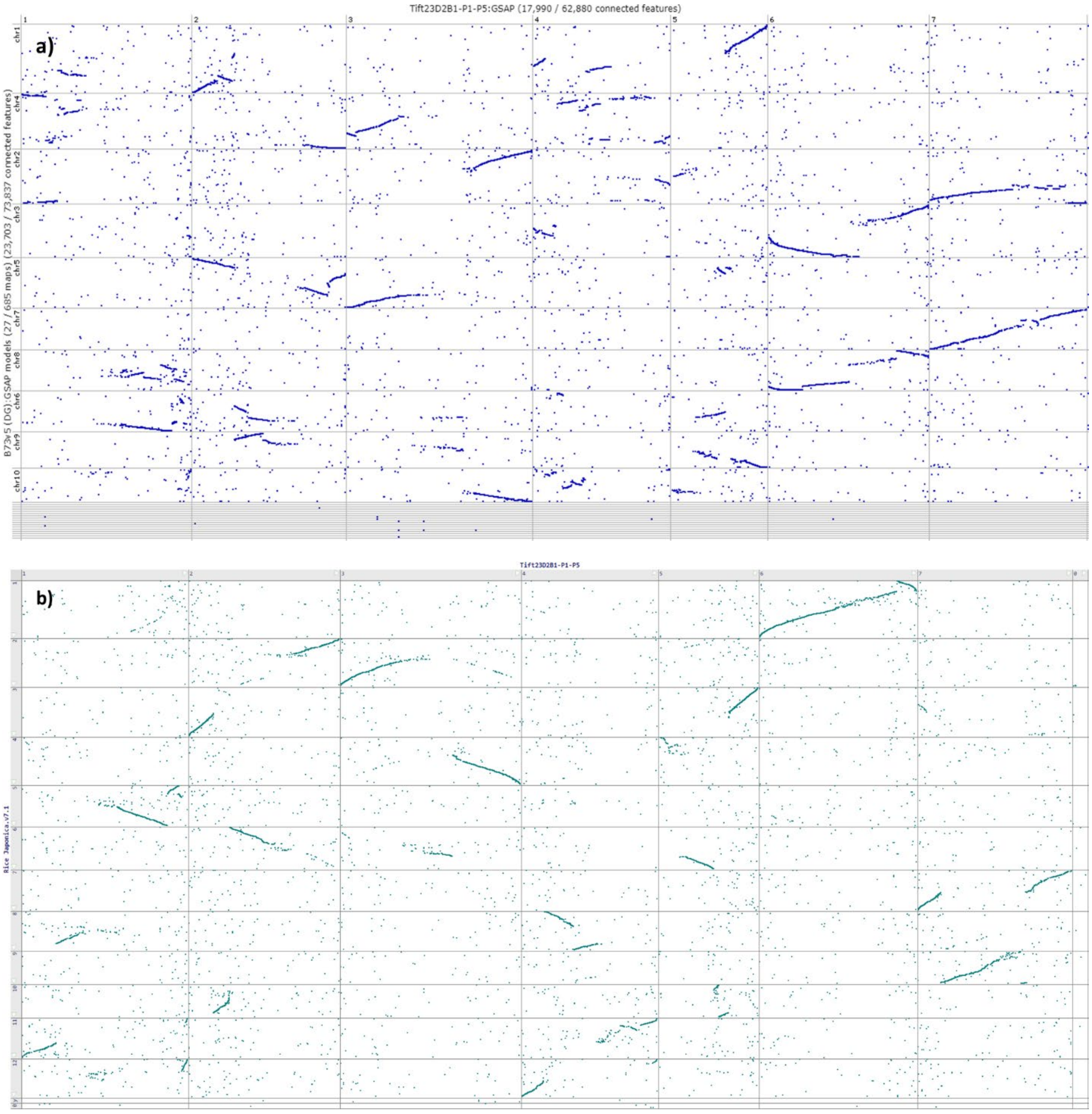

Continued………

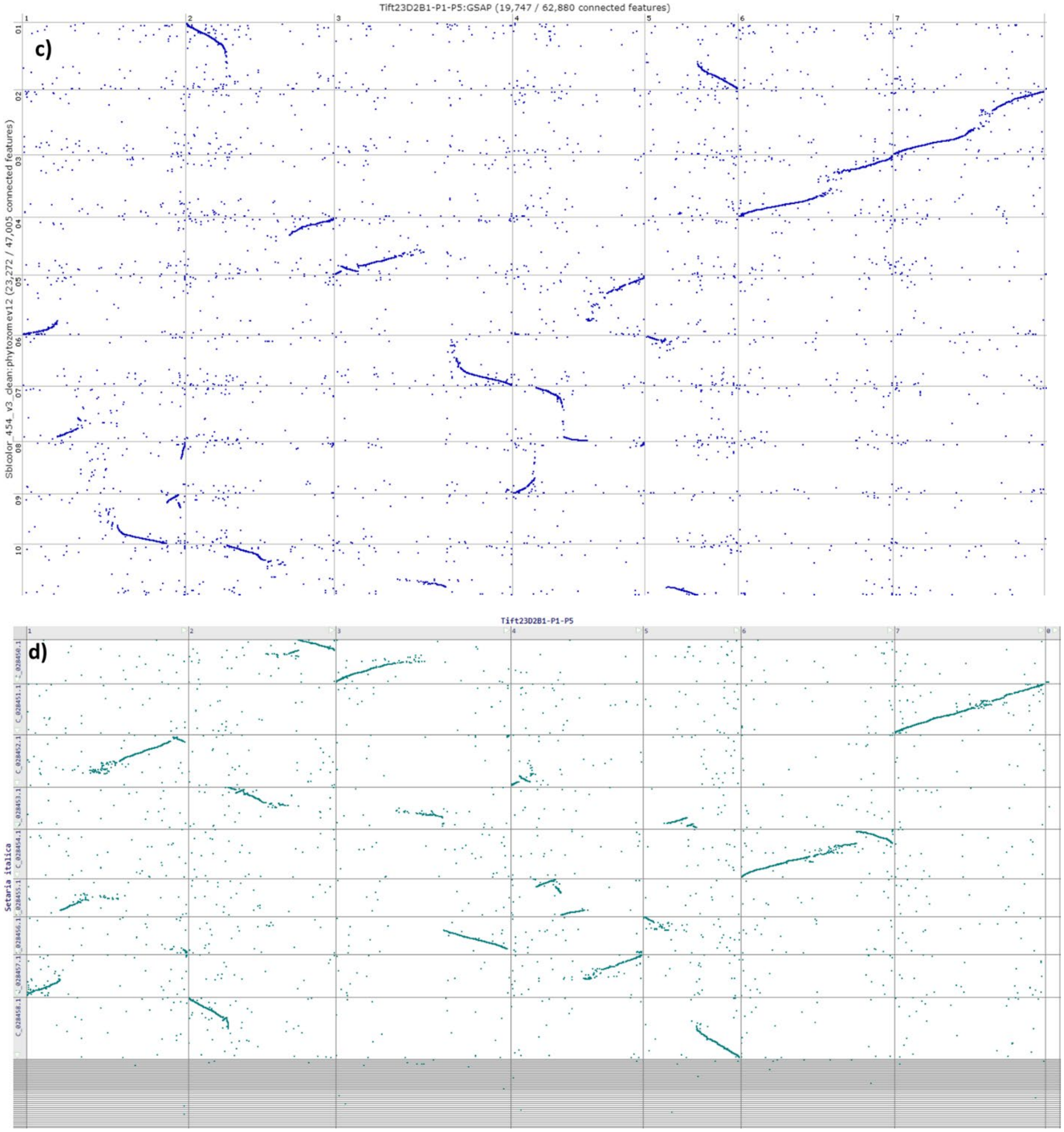

Supplementary Figure 9: Comparative genome mapping of millet with a) maize, b) rice, c) sorghum and d) foxtail millet. In all plots, x-axis is pearl millet genome.

Supplementary Figure 10:

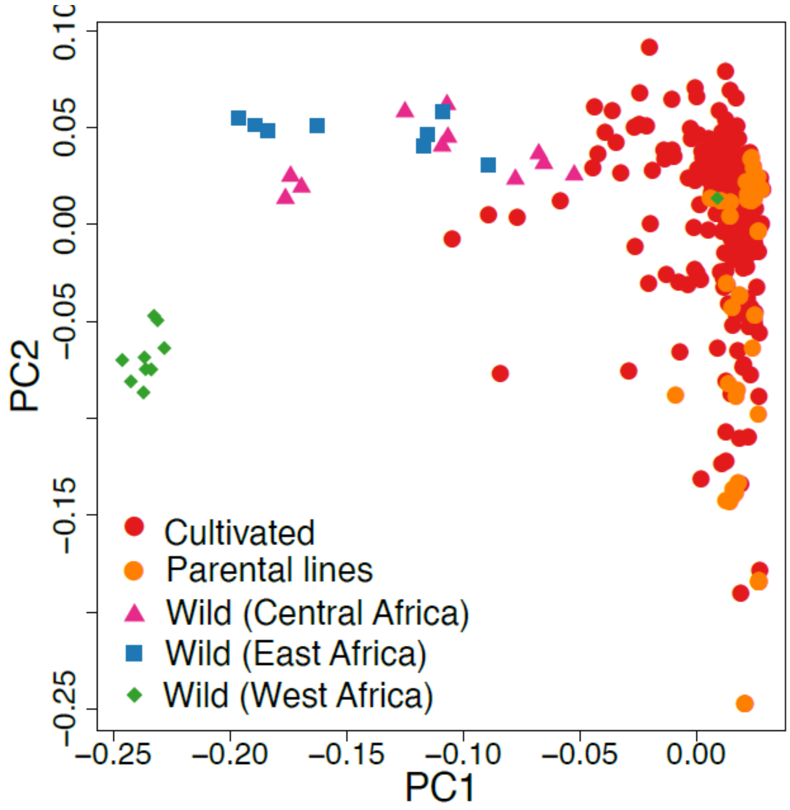

Supplementary Figure 10: Principal component analysis plot of 1028 pearl millet lines including pearl millet germplasm association panel (PMiGAP), B-lines and R-lines.

Supplementary Figure 11:

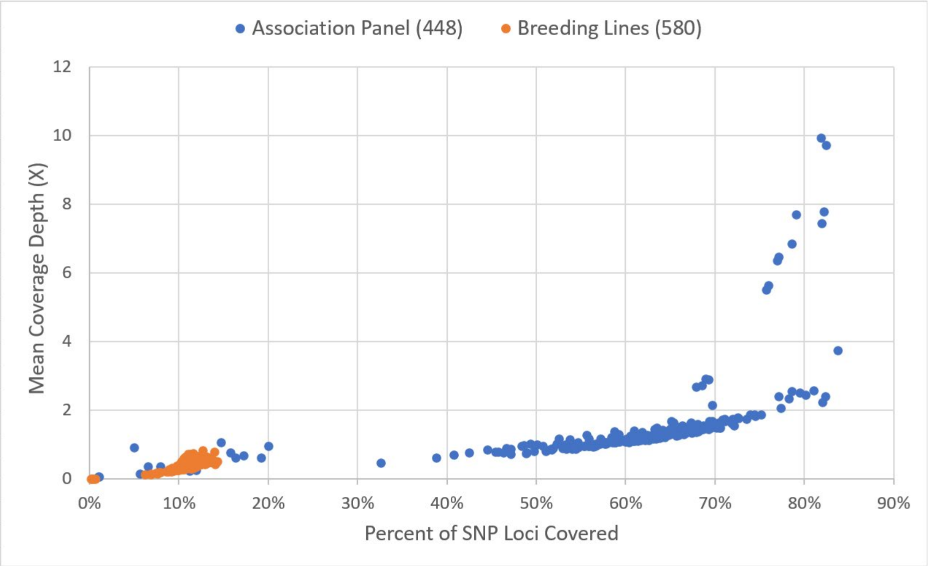

Supplementary Figure 11a: SNPs loci coverage of pearl millet germplasm. 448 lines from association panel PMiGAP (blue) were sequenced at 1 to 3X coverage depth and 20 lines were excluded from allele modelling due to a low coverage. All 580 breeding lines (B- and R-lines, orange) were sequenced at a super skim depth (<1X) and resulted in super low percent of SNP loci coverage, and thus excluded from allele modelling.

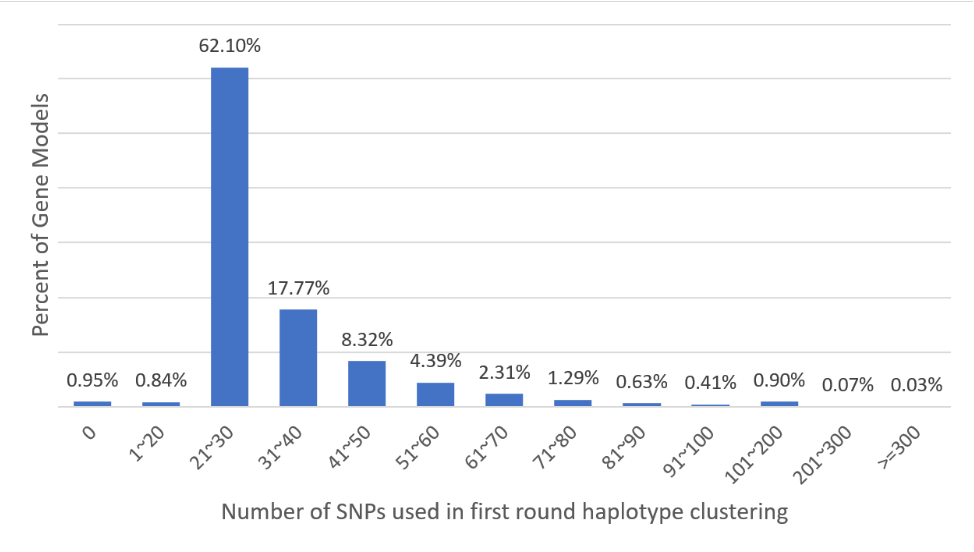

Supplementary Figure 11b: Distribution of the number of SNPs used in first round of clustering in Allele Modelling haplotyping of gene models. In the 36K TIFT filtered gene models set, 98.2% genes have more than 20 (minimum number of SNPs required for haplotype clustering) SNPs for PHASE1 haplotyping.

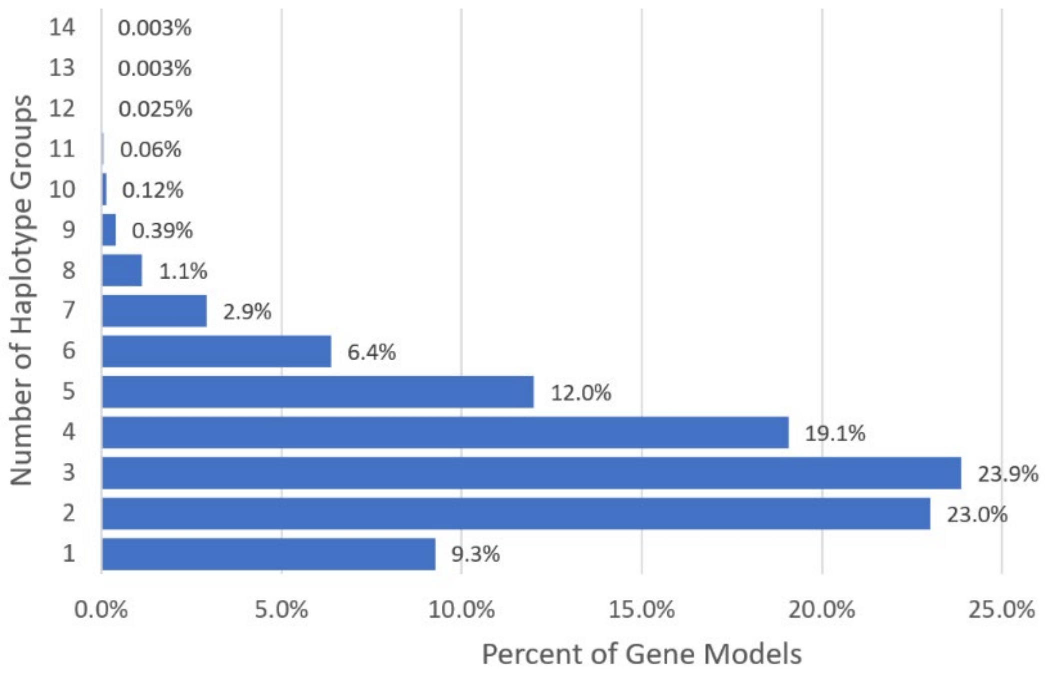

Supplementary Figure 11c: Number of haplotypes present at each locus.

Supplementary Table 1: List of rRNA and tRNA genes identified in Tift, ICMR 06777 and 843 B genomes.

ncRNA Categories	Tift	ICMR06777	843B	Tift2017
antisense_rna	4	0	5	NA
enzymatic_rna_gene	1	1	1	NA
group_ii_intron	25	27	47	NA
mirna_gene	184	208	164	157
ncrna_gene	2	2	2	NA
riboswitch	1	1	1	NA
rrna_gene	194	74	311	126
snorna_gene	295	274	290	551
snrna_gene	63	71	85	139
srp_rna_gene	7	0	0	NA
transcription_regulatory_region	5	4	4	NA
tRNA	713	532	705	879

Supplementary Table 2: List of fertility restoration/male sterility maintenance genes in 843B and ICMR 06777.

S.no	843B gene ID	ICMR 06777 gene ID	Percent Identity	Query coverage
	**Genes representing R- line pool**			
**1**	*dpca1g083060.842.1*	*dpca1g068620.841.1*	98.23	100
2	*dpca2g175040.842.1*	*dpca2g151750.841.1*	100	100
**3**	*dpca2g187160.842.1*	*dpca2g162810.841.1*	99.19	100
4	*dpca2g107790.842.1*	*dpca2g092840.841.1*	100	100
**5**	*dpca3g192470.842.1*	*dpca3g168130.841.1*	99.01	100
**6**	*dpca3g280390.842.1*	*dpca3g240910.841.1*	98.64	100
7	*dpca4g323310.842.1*	*dpca4g281020.841.1*	100	100
8	*dpca5g403200.842.1*	*dpca5g350470.841.1*	100	100
**9**	*dpca5g403200.842.1*	*dpca7g480100.841.1*	83.33	100
**10**	*dpca7g499590.842.1*	*dpca7g434840.841.1*	99.47	100
11	*dpca7g570070.842.1*	*dpca7g495100.841.1*	100	100
12	*dpca7g506860.842.1*	*dpca7g441680.841.1*	100	100
	**Genes representing B- line pool**			
13	*dpca6g423290.842.1*	*dpca6g370200.841.1*	100	100
14	*dpca3g212550.842.1*	*dpca3g186790.841.1*	100	100

Note: Genes from S.nos 1, 3, 5, 6, 9 and 10 showing polymorphism (percent identity differences) between ICMR 06777 and 843B are potential genes for male sterility maintenance and fertility restoration traits in pearl millet.
