# Supplementary material for: Improved pearl millet genomes representing the global heterotic pool offer a framework for molecular breeding applications
Source: Commun Biol. 2023 Sep 4;6:902. doi: 10.1038/s42003-023-05258-3 (PMC10477261; doi:10.1038/s42003-023-05258-3)
Supplement: Supplementary file 3 — Description of Additional Supplementary Files [file 42003_2023_5258_MOESM3_ESM.pdf]

Description of Additional Supplementary Files

File name: Supplementary Data 1
Description: Functional descriptions of genes present in the segmental inversion of chromosome 4 in Tift, ICMR 06777 and 843 B genomes.

File name: Supplementary Data 2
Description: Genome-wide association results for grain yield related traits in pearl millet.
